# Deposition of Magnetite Nanofilms by Pulsed Injection MOCVD in a Magnetic Field

**DOI:** 10.3390/nano8121064

**Published:** 2018-12-17

**Authors:** Anna Zukova, Arunas Teiserskis, Yuliya Rohava, Alexander V. Baranov, Sebastiaan van Dijken, Yurii K. Gun’ko

**Affiliations:** 1 School of Chemistry and CRANN Institute, Trinity College Dublin, Dublin 2, Dublin, Ireland; azukova@yahoo.com (A.Z.); arunotei@gmail.com (A.T.); 2 Education Department, Maxim Tank Belarusian State Pedagogical University, 18 Sovetskaya Street, 220050 Minsk, Belarus; yulitalia@mail.ru; 3 Information Optical Technology Centre, ITMO University, 197101 Saint Petersburg, Russia; a_v_baranov@yahoo.com; 4 Nanospin, Department of Applied Physics, Aalto University School of Science, P.O. Box 15100, FI-00076 Aalto, Finland; sebastiaan.van.dijken@aalto.fi

**Keywords:** magnetic nanofilms, magnetite, MOCVD, growth in magnetic field

## Abstract

This report is on the growth of Fe_3_O_4_ nanofilms on Al_2_O_3_(0001) and MgO(001) substrates with and without the presence of an external magnetic field using a pulsed injection metallorganic chemical vapour deposition (PI MOCVD) technique. The effects of growing magnetic oxide nanofilms in a 1 T field have been examined using various instrumental methods. It was found that the application of a magnetic field during PI MOCVD does not drastically alter the crystalline texture, surface morphology, and film thickness, but it significantly modifies the Fe_3_O_4_ film magnetisation and coercive field. Moreover, it was shown that the application of a 1 T field during the cooling of the sample also improves the magnetic properties. We believe that the large external field orients the magnetic spin structure at high temperatures (during growth or the initial stages of cool down) and that cooling through local magnetic ordering temperatures at Fe_3_O_4_ defect sites subsequently favours a ferromagnetic spin alignment. The control of magnetic properties of magnetite nanofilms by the application of magnetic fields during growth opens up new routes towards the fabrication and application of magnetic thin film devices.

## 1. Introduction

Magnetite (Fe_3_O_4_) is a popular magnetic oxide material that has been used in a large number of applications, including electric motors, electromagnets, transformers, video and audio adapters, magnetic inks, and biomedical techniques. Band structure calculations of Fe_3_O_4_ suggest that only spin down electrons are present at the Fermi level [1,2,3], which would imply that Fe_3_O_4_ is a half-metal. Although experiments have not confirmed the predicted half-metallicity, they do show that the number of spin down electrons is considerably larger than the number of spin up electrons at the Fermi level [4,5,6]. For this reason, Fe_3_O_4_ has recently attracted interest from the spintronics research field, where the nearly half-metallic character is utilised for efficient spin injection into semiconductors and sensitive magnetoresistance devices [7,8,9,10,11]. In addition, there is of great interest in the fabrication of magnetic nanofilms for potential applications in magnetic resonance imaging, electric motors, and magnetic hard disk drives for data storage [12,13,14]. 

Fe_3_O_4_ thin films have been grown on MgO [15,16,17,18,19,20,21,22], Al_2_O_3_ [15,16,17,20,23], Si [20,21,22,23,24,25,26], GaAs [23,27,28,29], and other substrates using various deposition techniques, including molecular beam epitaxy (MBE), pulsed-laser deposition (PLD), RF and reactive DC magnetron sputtering, and ion beam deposition (IBD). The observed magnetic properties of thin Fe_3_O_4_ films vary strongly in the literature due to differences in crystalline texture, stoichiometry, surface morphology, film strain, and lattice defects. One defect structure that particularly influences the magnetic response is the antiphase boundary which forms when neighbouring islands with different Fe_3_O_4_ symmetry coalesce during film growth. Coupling across antiphase boundaries can be ferromagnetic and antiferromagnetic and this leads to a highly complicated magnetic behaviour, including local out-of-plane moments, superparamagnetism, reduced film magnetisation, and saturation fields that can be two orders of magnitude larger than the anisotropy field of bulk Fe_3_O_4_ [30,31,32,33,34]. Antiphase boundaries are a direct result of the growth process, and the magnetic microstructure can, therefore, be altered by a variation of deposition parameters or substrate. Besides, it can be envisaged that the nature and strength of the magnetic coupling across antiphase boundaries can be tailored by the application of a magnetic field during film growth. Indications of the latter effect are an increase of magnetic remanence and a reduction of the saturation field when Fe_3_O_4_ films are magnetron sputtered in an applied magnetic field of 30–35 mT [35,36].

Previously, we have demonstrated that the application of a 1 T magnetic field during magnetite film growth significantly alters the magnetic properties without any visible changes in crystalline texture, surface morphology, and film thickness [37]. This anomalous magnetic behaviour was explained by the reduction in antiphase-boundary formation during film growth as it was confirmed by TEM analysis. Here, we report further research on the development of the growth of Fe_3_O_4_ films using pulsed injection metallorganic chemical vapour deposition (PI MOCVD) under different conditions. We also compare the deposition of crystalline magnetite nanofilms on Al_2_O_3_(0001) and MgO(001) substrates in a 1 T field and without a field.

## 2. Materials and Methods

### 2.1. Starting Materials, General Procedures, and Equipment

Single-crystal Al_2_O_3_(0001) (c-cut sapphire) and MgO(001) substrates from Crystal GmbH (Berlin, Germany) were used to deposit Fe_3_O_4_ films. The Al_2_O_3_ and MgO substrates were 5 × 5 × 0.5 mm^3^ in size and polished on both sides using a Logitech chemical mechanical polisher (CMP-Orbis System, Glasgow, UK), the polishing pad was made of polyurethane. After polishing the substrates, they were ultrasonically cleaned in acetone and ethanol and dried under vacuum. Anhydrous 1,2-dimethoxyethane 99.5% was purchased from Sigma-Aldrich (Dublin, Ireland) and used as a solvent for the metalorganic precursors in the PI MOCVD process. 99.9% purity Fe(SO_4_)_3_, NaCH_3_COO and *β*-diketone were also obtained from Sigma-Aldrich. Elemental analysis of the synthesised precursors was carried out at University College Dublin using an External Analytical CE-440 elemental analyser (EAI, Uxbridge, UK). A Pananalytical X’Pert Pro diffractometer (Malvern Panalytical Ltd., Royston, UK) with X’Celerator and PRS Pass detectors and a Cu cathode with a wavelength λ_Kα_ = 1.54056 Å was used to characterise the crystalline structure of the films. Raman spectra were measured with a Renishaw 1000 Micro-Raman system. This system consisted of an Ar^+^ ion laser (Laser Physics Reliant 150 Select Multi-Line, Edmund Optics, York, UK) with an excitation wavelength of 514.5 nm and a typical laser power of 3 mW. A white light interferometer was used to measure the film thickness. Prior to the measurements, the samples were masked with a patterned photoresist and then etched using HF or HCl acid which resulted in sharp film-substrate steps. The surface morphology of the samples was studied using a scanning electron microscope (variable pressure Hitachi S-3500N, Fukuoka, Japan). The volume of each deposited film was determined by multiplying the substrate area (5 × 5 mm^2^) by the thickness of the film. The mass of each deposited film was calculated by multiplying the volume by the density of the magnetite film, which was determined from X-ray reflectivity measurements using a Philips X’Pert Pro diffractometer, Philips Analytical, Royston, UK). Magnetic measurements were performed using a Quantum Design MPMS XL SQUID (Quantum Design, Surrey, UK) magnetometer. The magnetic hysteresis curves were measured up to a field of 1 T at a temperature of 300 K. 

### 2.2. Synthesis of Fe(tmhd)_3_ Precursor

Fe_2_(SO_4_)_3_ and an excess of NaCH_3_COO (ratio 1:2) were first dissolved in distilled water. An ethanol solution of the *β*-diketone was then added to the aqueous Fe_2_(SO_4_)_3_ solution. The Fe(tmhd)_3_ was removed by filtration and recrystallised from hexane. The melting point of Fe(tmhd)_3_ is 163 °C. Analysis calculated for Fe(tmhd)_3_ (FeC_33_H_57_O_6_): C, 65.44; H, 9.51; N, 0; indicated: C, 65.52; H, 9.52; N, 0. 

### 2.3. Pulsed Injection Metallorganic Chemical Vapour Deposition (PI MOCVD) Reactor 

**Figure 1:**
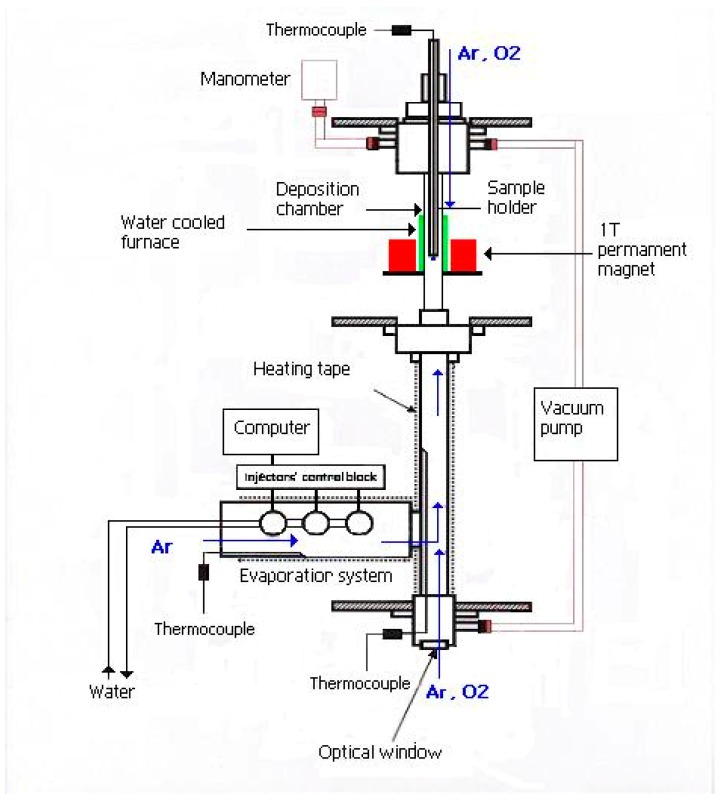
Schematic illustration of the PI MOCVD reactor with a 1 Tesla magnet.

A schematic illustration of the home-made PI MOCVD reactor with a 1 T switchable magnet is shown in Figure 1. The reactor consisted of a water-cooled furnace that was placed inside the 8-cm bore of the magnet. A quartz tube with a diameter of 3.5 cm was located inside the furnace and served as the deposition chamber for substrates. The precursor was kept at room temperature and small droplets of it were sequentially pulsed into the hot evaporation chamber by an electromagnetic valve. The evaporation chamber was heated to a temperature of 140 °C, which is sufficiently high to rapidly evaporate the injected precursor, but low enough to avoid precursor decomposition. Ar carrier gas was subsequently used to transport a small amount of evaporated precursor to the deposition chamber, in which thermal decomposition and thin film growth occurred at a well-controlled substrate temperature. The applied magnetic field was oriented perpendicular to the substrate plane.

## 3. Results and Discussion

### 3.1. PI MOCVD of Fe_3_O_4_ Films 

Weakly magnetic α-Fe_2_O_3_ is the most stable iron oxide phase below 560 °C. As a result, this phase tends to dominate iron oxide films that are grown in a reactive oxidising atmosphere. The formation of Fe_3_O_4_ often requires an additional annealing step at temperatures below 350 °C for 3–5 h in a reduced H_2_ atmosphere. In our experiments, direct growth of iron oxide films with a pure Fe_3_O_4_ phase was performed using PI MOCVD with an inert argon or argon and hydrogen (90%Ar+10% H_2_) as carrier gases. The gas flow was 80 mL/min with the reactor pressure of 5 mbar. For this single-step reaction, we used an iron(III) tetramethylheptanodionate (Fe(tmhd)_3_) precursor and Al_2_O_3_(0001) and MgO(001) substrates. 

To facilitate the application of a large magnetic field, a new PI MOCVD reactor was constructed inside a switchable 1 T magnet as schematically illustrated in Figure 1. This new reactor was also used to grow films without an external magnetic field. After deposition the films were slowly cooled in vacuum to room temperature. For a constant number of deposition pulses (1000), a Fe_3_O_4_ film thickness varying from about 20 nm to 140 nm was obtained depending on the selected temperature and substrate. This large sensitivity of the deposition rate reflects different optimal precursor decomposition/absorption and diffusion rates on Al_2_O_3_(0001) and MgO(001). Moreover, some variation in film thickness might be explained by gaseous phase turbulences that move the deposition centre away from the substrate. Therefore, to avoid gaseous phase turbulences we used an optimal gas flow of 80 mL/min in our experiments. 

It has previously been demonstrated that the PLD technique enables the preparation of high quality stoichiometric metal oxide films such as magnetite [17,24], aluminum-doped ZnO (AZO) [38], and even the incorporation of metal (Au) nanoparticles in stoichiometric TiO_2_ films [39], because the stoichiometry of the target can also be maintained in the deposited films. However, the growth of high-quality films by conventional MOCVD requires volatile precursors that will undergo pyrolysis easily. The low thermal stability of the metalorganic precursors due to their polymerisation or hydrolysis might change the rate of evaporation. This can cause difficulties in controlling the composition of the vapour phase and stoichiometry of films. To avoid that we use the PI MOCVD technique where the growth rate, thickness, and stoichiometry of the film can be controlled precisely by changing the concentration of the solution, number of pulses, pulse duration, and injection frequency. This was especially important for the deposition of Fe_3_O_4_ nanofilms in our work. Table 1 summarises the deposition conditions and resulting Fe_3_O_4_ film thickness. 

### 3.2. Structural Characterisation 

#### 3.2.1. X-ray Diffraction Studies of Fe_3_O_4_ Films on Al_2_O_3_(0001)

**Figure 2:**
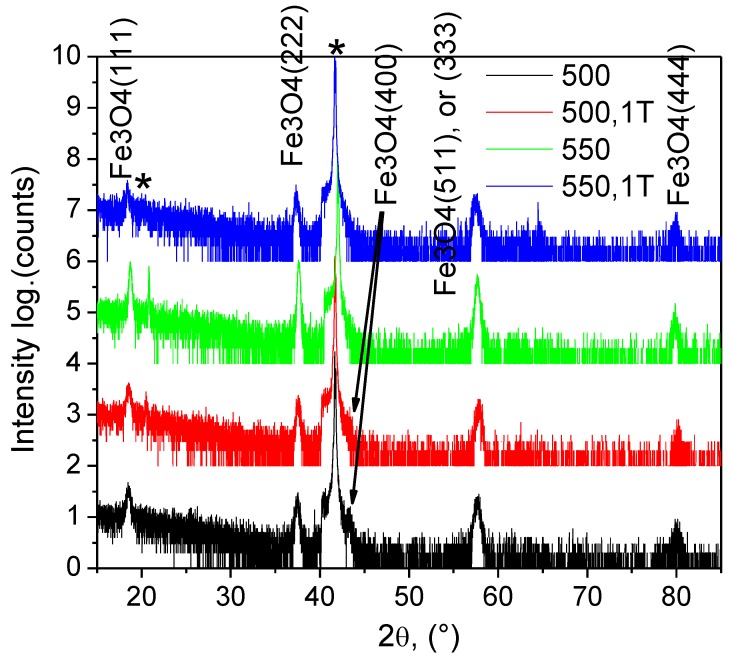
XRD patterns of the Fe_3_O_4_ films grown on Al_2_O_3_ sapphire in Ar+H_2_ atmosphere (*-Al_2_O_3_ substrate peaks).

Structural characterisation of the films was conducted using a Pananalytical X’pert Pro diffractometer with a Cu *K_α_* source. Figure 2 shows *θ*–2*θ* X-ray diffraction scans of iron oxide films that were grown on Al_2_O_3_(0001) at different temperatures and applied magnetic field. The main film peaks correspond to the (111) planes of the Fe_3_O_4_ cubic inverse spinel lattice. No other Fe_3_O_4_ reflections were observed indicating highly-oriented film growth irrespective of deposition temperature. The full width at half maximum of the (333) peak is about 0.6° for all films. Using Scherrer’s formula this corresponds to an average grain size of about 15 nm. The out-of-plane lattice parameter as experimentally determined from the position of the (333) reflection, is about 8.335 ± 0.005 Å for T = 450 °C and 500 °C, and 8.305 ± 0.005 Å and 8.285 ± 0.005 Å for T = 550 °C and 600 °C, respectively. This is slightly smaller than the Fe_3_O_4_ bulk lattice constant of 8.397 Å and, thus, the nanofilms are compressed perpendicular to the film plane. The small deviations in the lattice parameters of the Fe_3_O_4_ films from single crystal bulk samples are mostly due to substrate-induced strain. In addition to the clear Fe_3_O_4_ reflections, a diffraction peak is also measured at 39.3° on films grown at 450 °C, 500 °C, and 550 °C. This reflection, which is most intense for the 500 °C data, corresponds to weakly magnetic α-Fe_2_O_3_. The presence of a minority α-Fe_2_O_3_ phase is also confirmed by the colour of these films which is brown-red rather than metallic dark, a relatively large electrical resistance and Raman spectra. In contrast, the X-ray diffraction scan of the film grown on Al_2_O_3_(0001) at 600 °C does not show any evidence of other iron oxide phases besides Fe_3_O_4_. Moreover, these films have metallic dark in colour, exhibit a resistivity that is typical for Fe_3_O_4_ at room temperature, and despite they are thinner than the other films, it’s Fe_3_O_4_ (111) reflections are the most intense. It was found that the application of a 1 T magnetic field during PI MOCVD growth of Fe_3_O_4_ on Al_2_O_3_(0001) has a negligible effect on the structural properties of the films as illustrated by the *θ*–2*θ* X-ray diffraction scans of Figure 2 and Figure S1 in the electronic supporting information (ESI). A comparison between the films grown and cooled with (g + c), grown without and cooled with (c, only at 500 °C and 550 °C,) and grown and cooled without a 1 T magnetic field does not reveal any clear differences. In all cases, the film are highly oriented with a preferred Fe_3_O_4_ (111) film texture and a minority α-Fe_2_O_3_ phase for nanofilms grown at 450 °C, 500 °C, and 550 °C. Also, the out-of-plane lattice parameter and grain size do not change upon the application of a magnetic field during PI MOCVD. This result contrasts with experiments carried out by Tang et al. [36] which demonstrated that a 35 mT magnetic field alters the preferred crystalline orientation of magnetron-sputtered Fe_3_O_4_ films. The different nature of the physical and chemical vapour deposition techniques is most likely responsible for this difference. 

The average grain size was calculated from XRD (333) reflection for Fe_3_O_4_ using Scherrer’s formula. We found that average grain size of Fe_3_O_4_ films grown at different temperatures in Ar did not change very drastically and was lying in the interval of 12–18 nm. The slightly smaller grain sizes were found for the films grown in Ar+H_2_ reactive atmosphere. At the same time the films grown with different pulse numbers had larger grain size. However, the results we obtained using the Fe_3_O_4_ (111) peak rather than the (333) peak. The average grain sizes for all Fe_3_O_4_ films grown on Al_2_O_3_ substrate are shown in Table S1 (ESI).

#### 3.2.2. X-ray Diffraction Studies of Fe_3_O_4_ Films on MgO(001)

**Figure 3:**
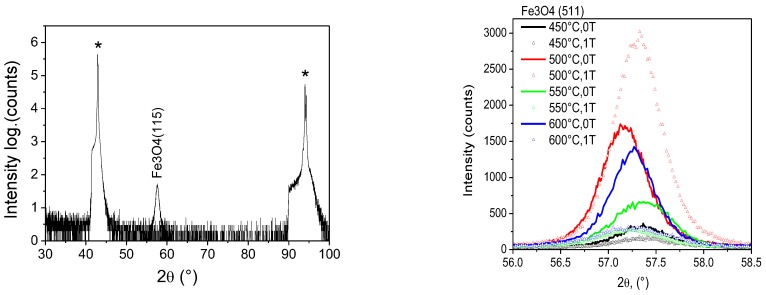
(**Left**) Typical XRD pattern of a Fe_3_O_4_ film on a MgO(100) substrate. (**Right**) Fe_3_O_4_ (511) reflections for the films grown at different temperature in and without external magnetic field (*-MgO substrate peaks).

**Figure 4:**
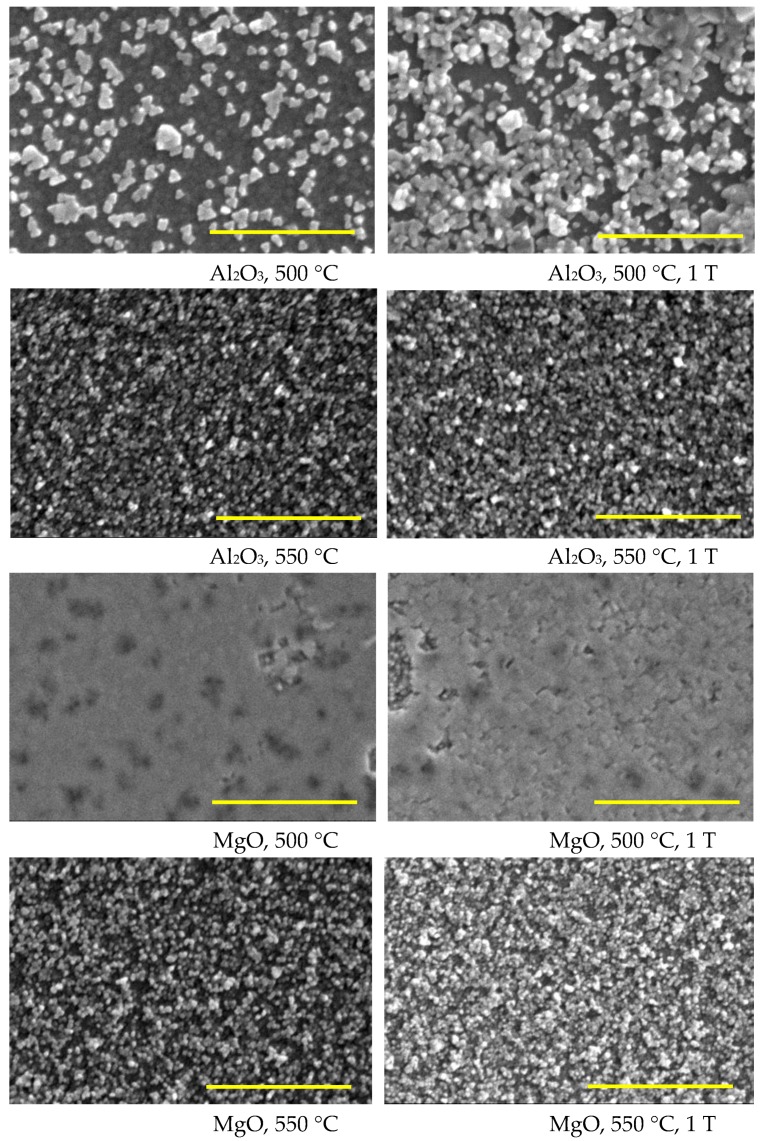
SEM micrographs of the Fe_3_O_4_ films grown in Ar+H_2_ atmosphere. All scale bars are 1 µm.

Previously, highly-oriented and fully-epitaxial Fe_3_O_4_ films have been grown on MgO(001) using different deposition techniques [15,16,17,18,19,20,21,22]. For this system, the lattice constant of Fe_3_O_4_ (*a* = 8.397 Å) is nearly twice as large as that of MgO (*a* = 4.212 Å) and, thus, the lattice mismatch between film and substrate is relatively small. Consequently, it is impossible to detect the Fe_3_O_4_(004) reflection at an expected angle of 43.052° in a standard *θ*–2*θ* X-ray diffraction scan as it overlaps with the more intense MgO(002) substrate peak at 42.916°. For this reason, X-ray diffraction analysis of Fe_3_O_4_ films on MgO(001) was conducted by tilting the sample at a *psi* angle of 15.79°, which corresponds to the angle between the (001) and (115) lattice planes. In this configuration, the *θ*–2*θ* scans exhibit a single Fe_3_O_4_(115) reflection. The typical XRD pattern of a Fe_3_O_4_ film on a MgO(100) substrate, as well as Fe_3_O_4_ (511) reflections for the films grown at different temperature in and without external magnetic field are presented in Figure 3. The Phi scan of the (511) reflection for a Fe_3_O_4_ film on MgO(100) is shown in Figure S2 (ESI). The measurements clearly show that all films are single crystalline irrespective of the deposition temperature. The small shift in the position of the (115) reflection and thus the amount of film strain is non-monotonous in temperature and this is most likely due to the large variation in film thickness as listed in Table 1. The average FWHM of the (115) diffraction peak amounts 0.6° and this corresponds to an average grain size of about 15 nm. Figure 4 shows a phi scan of the Fe_3_O_4_(115) reflection. Four peaks of similar intensity are observed when the sample is rotated by 360°. This is a typical result for fully epitaxial films with an in-plane Fe_3_O_4_(100)/MgO(100) alignment. In contrast to Fe_3_O_4_ on Al_2_O_3_(0001), the films on MgO(001) do not contain α-Fe_2_O_3_. Additionally, all films are grey metallic in colour and their resistivity corresponds closely to that of Fe_3_O_4_ at room temperature. 

The average grain size calculated for the films had similar values as calculated for the films grown on the Al_2_O_3_. No dependence between deposition temperature and particle size was found for the Fe_3_O_4_ films grown on MgO substrate. All results for Fe_3_O_4_ films grown on MgO substrate are summarised in the Table S2 (ESI). Films grown in 1 T external magnetic field have shown slightly larger FWHM values than those grown without magnetic field. The average grain size calculated for the films grown in an external magnetic field is also slightly larger than those for the films grown without a magnetic field. We do not exclude the possibility that the external magnetic field applied during the growth of the films is responsible for the observed changes in FWHM and crystallite size in PIMOCVD-grown Fe_3_O_4_ films. 

As it was observed for films on Al_2_O_3_(0001), the application of a magnetic field does not drastically alter the crystalline texture of Fe_3_O_4_ on MgO(001). The film strain and average grain size are similar for Fe_3_O_4_ films grown with and without a 1 T magnetic field during PI MOCVD growth as illustrated by the negligible changes in the position and width of the (115) reflection.

### 3.3. Surface Morphology 

It has been previously reported that the surface microstructure of superconducting oxide films changes significantly upon the application of a magnetic field during MOCVD growth [40]. To monitor the influence of the magnetic field, deposition temperature, and substrate, the surface morphology of the Fe_3_O_4_ films was imaged using scanning electron microscopy (SEM). SEM images of the surface microstructure of the magnetite films grown in Ar+H_2_ atmosphere are shown in Figure 4. Films grown at 550 °C were composed of approximately ~25–35 nm aggregated grains. However, the films on *c*-cut sapphire grown at 500 °C had a different microstructure and consisted of the larger (~80 nm) aggregates distributed over the smooth matrix. The different surface microstructure for these films can be easily explained by the fact that the films have different XRD reflection (Fe_3_O_4_ (400)) not observed in other films, which results in this particular microstructure. Generally, films grown on Al_2_O_3_ substrate in Ar only and Ar+H_2_ atmosphere at 500 °C have a much rougher surface consisting of randomly-oriented grains than films grown at other deposition temperatures. We believe that this structure is a result of the high saturation of precursor vapours near the substrate surface and substrate temperature of 500 °C. This results in the rapid supply of reactants, but the lower substrate temperature limits surface diffusion, thus resulting in fewer nucleation sites.

At the same time, the surface of the films grown on MgO substrate at 500 °C consisted of coalescent particles, similar to that found for the films grown at 500 °C in Ar atmosphere on MgO substrates.

SEM images of the samples grown in pure argon atmosphere are shown in ESI (Figures S3 and S4) The SEM images in Figure S3 clearly demonstrate that the surface microstructure of PI MOCVD-grown Fe_3_O_4_ films on Al_2_O_3_(0001) varies considerably with deposition temperature, but not with the applied magnetic field. The surface of the film grown at 450 °C resembles an aggregation of grains with an average size of about 20 nm. A similar surface morphology is also obtained at 550 °C and 600 °C, but at these temperatures the grains form smaller aggregates. In general, the Fe_3_O_4_ films on Al_2_O_3_(0001) are relatively rough, which might be caused by grain defects, such as small angle boundaries. The film grown at 500 °C exhibits the largest surface roughness and its surface contains many crannies, which may be due to the thermal stress. It is believed that the boundary region may contain some voids, which would definitely increase the electrical resistivity of the films. In fact, the multimeter measurements proved that films grown at 500 °C had resistivity over 5 MΩ. This disordered surface morphology correlates with a large film resistivity, which is also associated with a high density of film defects and the formation of a secondary α-Fe_2_O_3_ phase. 

Figure S4 (ESI) shows SEM images of Fe_3_O_4_ films on MgO(001). Again, no clear magnetic field effects are observed. The film grown at 450 °C is smooth and its surface consists of small particle aggregates with an average size of 25–35 nm. The films that were grown at 550 °C and 600 °C possess a similar surface morphology with slightly larger aggregates. The deposition of Fe_3_O_4_ films on MgO(001) at a temperature of 500 °C results in a rather different surface microstructure. In this case, the film consists of coalesced particles, but contrary to Fe_3_O_4_ films grown on Al_2_O_3_(0001) at the same temperature, it now exhibits the much lower electrical resistivity of ~50 kΩ. 

The absence of any observable magnetic field effects in the SEM measurements agrees with the X-ray diffraction analysis. We therefore conclude that the application of a large 1 T magnetic field during PI MOCVD growth of Fe_3_O_4_ does not have a major influence on the crystalline texture and surface morphology of the films. 

**Figure 5:**
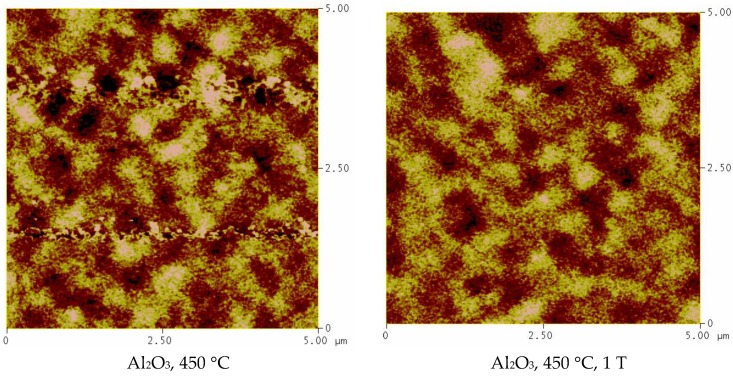

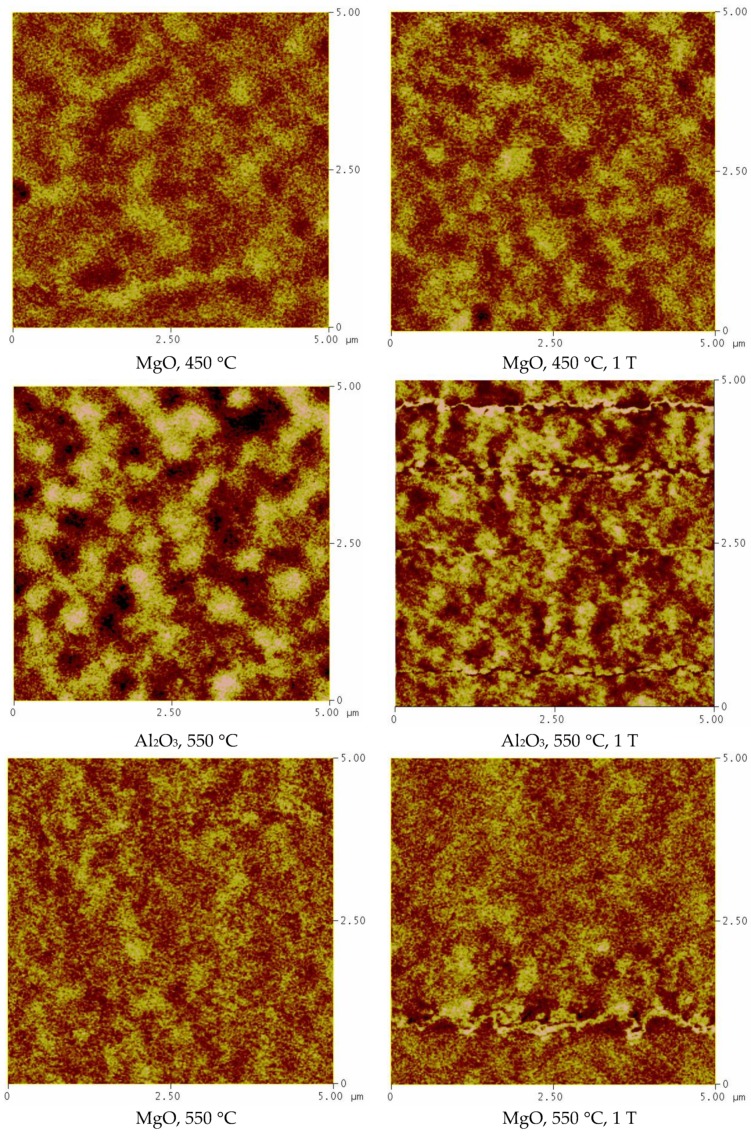
MFM images for the Fe_3_O_4_ films grown in different conditions. The dimensions of the squares are 5 μm.

The magnetic domain structure of the Fe_3_O_4_ films was also investigated by magnetic force microscopy (MFM). The MFM measurements revealed that films have an irregular domain structure. J. Cheng et al. previously reported that the domain structure morphology of heteroepitaxial Fe_3_O_4_ films grown by MBE strongly depends on the substrate [41]. The researchers found that the films grown on the SrTiO_3_ substrate, which has the largest lattice mismatch with Fe_3_O_4_, have a nanoscale domain size of ~200 nm. However, when grown on MgO, the films had the largest domain size. In contrast to the observed domain size increase with the decrease of the lattice mismatch between Fe_3_O_4_ and the substrate, our PI MOCVD grown magnetite films had a similar domain structure when grown on *c*-cut sapphire and MgO substrates. The films deposited in Ar atmosphere on Al_2_O_3_ substrate at 450 °C showed well-defined magnetic domains of approximately ~400–500 nm in size. The films grown at 450 °C on MgO substrate had a similar domain structure, however, it was not that well defined as on *c*-cut sapphire. The films retained the irregular shape domain structure with approximately 400–500 nm then grown in different conditions, suggesting that growth parameters, such as deposition temperature (550 °C) or reactive atmosphere (Ar+H_2_), do not have a major impact on the domain structure. The structure of the magnetic domains did not change either in size or shape when the external magnetic field was applied during the growth of the films. The MFM images for the Fe_3_O_4_ films are shown in Figure 5.

### 3.4. Raman Spectroscopy

Room temperature Raman spectroscopy was used to confirm the predominance of the Fe_3_O_4_ phase and to detect the presence of any other possible iron oxide phases. The Ar^+^ ion laser source (Laser Physics Reliant 150 Select Multi-Line, Edmund Optics, York, UK) in the Raman spectrometer had an excitation wavelength of 514.5 nm and a maximum power of about 10 mW. In order to avoid excessive heating and oxidation, a reduced laser power of 3 mW was used during all measurements. 

**Figure 6:**
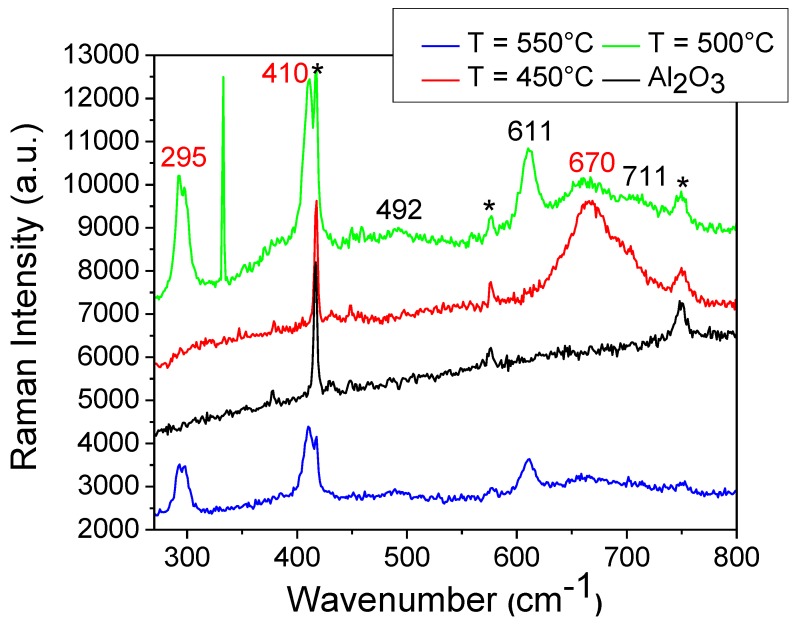
Raman spectra of Fe_3_O_4_ films on Al_2_O_3_(0001), where * indicates substrate peaks, numbers in red indicate the approximate peak positions of the Fe_3_O_4_ phase and the numbers in black indicate α-Fe_2_O_3._

Generally, magnetite has four actives modes, namely A_1g_, E_g_, and 3T_2g_ which are Raman active, and 4T_1u_, which is an infrared active mode. The A_1g_ mode is the highest frequency mode occurring at 670 cm^−1^. The E_g_ mode occurs at 306 cm^−1^ and the T_2g_ modes occur at 193 cm^−1^ (T^1^_2g_), 540 cm^−1^ (T^2^_2g_), and 490 cm^−1^ (T^3^_2g_) [42]. The magnetite nanofilms grown on Al_2_O_3_ substrates showed Raman shifts (A_1g_) corresponding to the Fe_3_O_4_ phase at ~667, 663, and 669 cm^−1^ for films grown at 450, 500, and 550 °C, as illustrated by the spectra of Figure 6. The A_1g_ mode is directly linked to the structure of the magnetite system [43] and possesses a minimum FWHM for the film grown at 450 °C, suggesting a better crystalline quality for this film. The Raman bands are shifted to slightly lower wave numbers in comparison with those observed for bulk Fe_3_O_4_ (670 cm^−1^), which can be explained by in-plane tensile film strain [44]. The T^3^_2g_ mode is observed only for films grown at 500 °C and 550 °C at ~492 cm^−1^, while the E_g_ mode is not observed at all. There is also a peak at 297 cm^−1^, which suggests the beginning of oxidation as shown by Dunnwald and Otto [45] and Hart et al. [46], especially when measured in combination with additional lines at 410–420 cm^−1^. In fact, the Raman spectra of our films show two peaks at ~410 cm^−1^ and 711 cm^−1^ (the peak at 711 cm^−1^ is observed for all films, while the peak at 410 cm^−1^ is measured only on films grown at 500 °C and 550 °C). These peaks are a characteristic signature of α-Fe_2_O_3_ [47,48].

The Raman measurements therefore confirm that the Fe_3_O_4_ films on Al_2_O_3_(0001) consist of a predominant magnetite phase with the inclusion of a secondary α-Fe_2_O_3_ phase in films that were prepared at 450 °C, 500 °C, and 550 °C. The results obtained by Raman spectroscopy are in line with the XRD analysis data of the Fe_3_O_4_ films. 

### 3.5. Magnetic Properties

The magnetic properties of the films were studied using a superconducting quantum interference device (SQUID). Magnetic characterisation of the Fe_3_O_4_ films was carried out at 300 K in a maximum in-plane magnetic field of 1 T. The hysteresis curve of some of the Fe_3_O_4_/MgO(001) samples were also measured using an out-of-plane magnetic field. The reported values for the saturation magnetisation (M_S_) and remanent magnetisation (M_R_) are approximate values, as the raw measurements also contain a diamagnetic contribution from the substrate and an uncertainty in the film thickness of about 5%. The substrate contribution was determined by fitting the high-field data to a straight line and this was subsequently subtracted from the experimental data to recover the hysteresis loops of the Fe_3_O_4_ films. 

#### 3.5.1. Fe_3_O_4_ Films on Al_2_O_3_ Substrates

**Figure 7:**
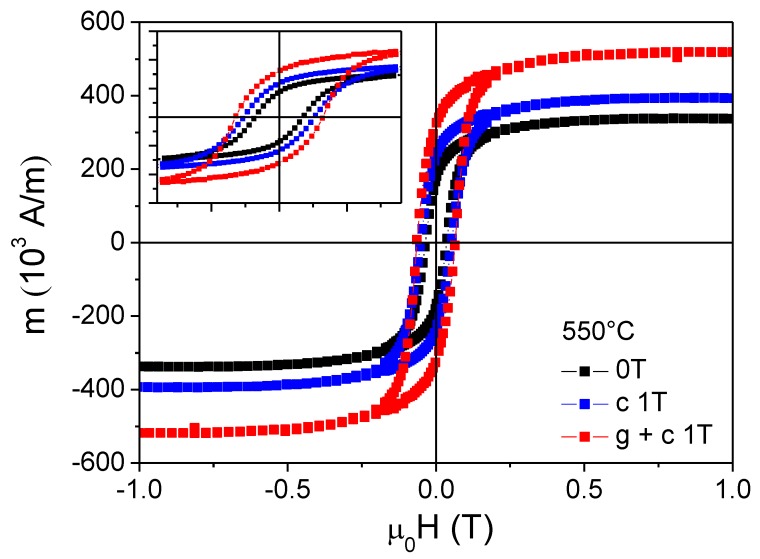

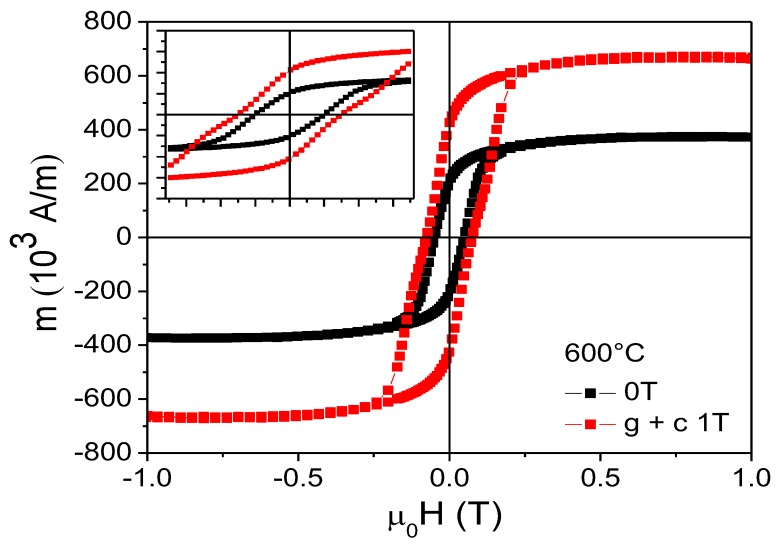
Hysteresis curves of Fe_3_O_4_ films grown on Al_2_O_3_ substrates at 550 °C (top) and 600 °C (bottom) with and without an external magnetic field of 1 T. The blue data points indicate the effect of cooling in an external magnetic field of 1 T.

Magnetic hysteresis curves of Fe_3_O_4_ films on Al_2_O_3_(0001) are rounded and tilted, as illustrated by the SQUID data in Figure 7. The magnetic switching field was 36 mT and 50 mT for films grown at 550 °C and 600 °C, respectively, and the film magnetisation saturations were about 0.5 T. Moreover, the in-plane remanent magnetisation is only 50–60% of its saturation value. Table 2 summarises the magnetic properties of all films. The data clearly demonstrate a strong dependence of the Fe_3_O_4_ film magnetisation on deposition temperature. The saturation magnetisation for films grown at 450 °C is only 227 × 10^3^ A/m, which is considerably smaller than the bulk value of 471 × 10^3^ A/m [49]. However, larger saturation values are obtained for films grown at 550 °C (M_S_ = 337 × 10^3^ A/m) and 600 °C (M_S_ = 372 × 10^3^ A/m). The PI MOCVD result for T = 600 °C is within the range of reported magnetisation values (370 − 470 × 10^3^ A/m) for sputtered and pulsed-laser deposited thin Fe_3_O_4_ films on MgO and Si substrates [15,16,17,18,19,20,21,22,23,24,25,26]. The observed increase of saturation magnetisation with deposition temperature correlates with a reduction of the minority α-Fe_3_O_4_ phase as inferred from X-ray diffraction analysis and Raman spectroscopy. 

**Figure 8:**
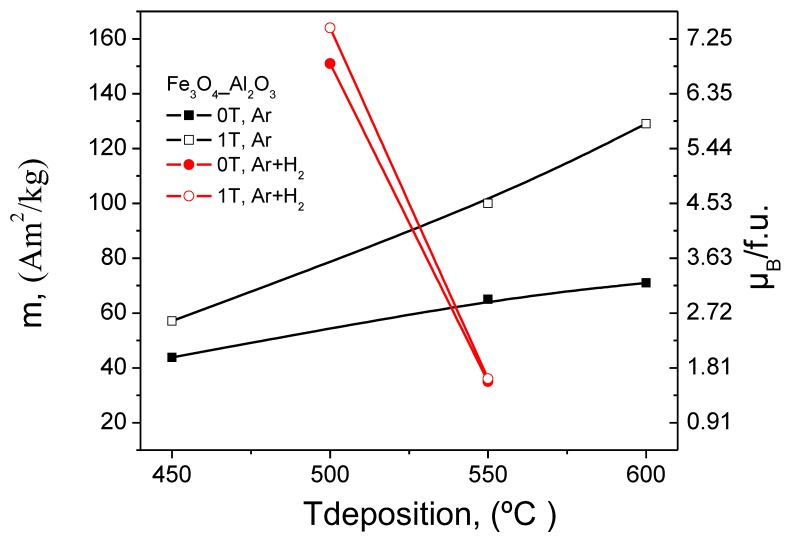
Graphical representation of the M_S_ and μ_B_/f.u. values versus deposition temperature for the films on Al_2_O_3_ grown in and without external magnetic field.

The data in Figure 8 and Table 2 reveal large magnetic field effects during PI MOCVD growth of Fe_3_O_4_ on Al_2_O_3_(0001). The application of a 1 T field resulted in increases of the coercivity and film magnetisation over the entire temperature range. The largest effects were obtained at 600 °C where PI MOCVD in a 1 T field increases the saturation magnetisation by 80% and more than doubles the remanence of Fe_3_O_4_ films. The magnitude of the field-induced modifications was unprecedented and somewhat surprising as they do not correlate with clear structural changes. The magnetic field thus drastically alters the magnetic microstructure without major changes in crystalline texture, surface morphology, and film thickness. To test if an enhancement of film magnetisation is only obtained during Fe_3_O_4_ film growth, we also grew some films without applied magnetic field and subsequently cooled them down to room temperature in 1 T. In this case, a smaller yet significant increase of the remanent and saturation magnetisation was observed. This clearly illustrates that field-induced changes in the magnetic microstructure are at least partially thermally activated and independent of the MOCVD growth process. 

#### 3.5.2. Fe_3_O_4_ Films on MgO Substrates

**Figure 9:**
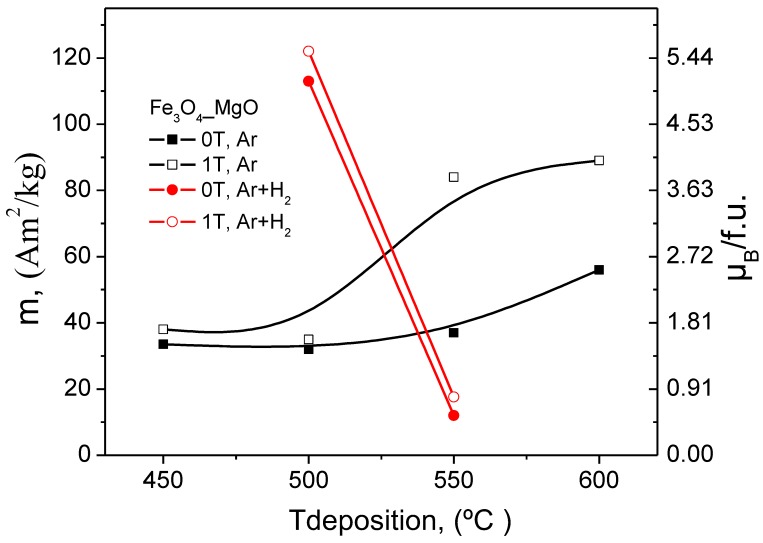
Graphic presentation of the M_S_ and μ_B_/f.u. values versus deposition temperature for the films on MgO, grown in and without external magnetic field.

The magnetic properties of Fe_3_O_4_ films on MgO(001) are qualitatively similar to those on Al_2_O_3_(0001). This is demonstrated by the data in Table 3 and the magnetic hysteresis curves (see Figure S5). To better observe the changes in the magnetisation values with deposition temperature for the films grown in and without external magnetic field the results have been plotted and shown in Figure 9. Common features include a small remanence, an increase of the film magnetisation with deposition temperature, and anomalous magnetic field effects during PI MOCVD growth. In addition to the many similarities, there are also some differences. First of all, the magnetic switching field is considerably smaller for Fe_3_O_4_ films on MgO(001). This is at least partly explained by the much smoother Fe_3_O_4_ surface morphology on MgO substrates. Second, the Fe_3_O_4_ saturation field of ~0.7 T on MgO is larger than on Al_2_O_3_—this might be due to the presence of antiphase boundaries. This structural defect, which is a common feature in Fe_3_O_4_/MgO(001) [30,31,32,33,34], is generally held responsible for a highly complicated magnetic behaviour including local out-of-plane moments, reduced film magnetisation, and large saturation fields. SQUID magnetisation measurements in a perpendicular applied magnetic field indeed confirm the presence of a considerable out-of-plane remanence and together with a relatively small saturation magnetisation this further indicates a high degree of magnetic disorder. 

The application of a magnetic field during PI MOCVD growth drastically influences the magnetic microstructure of Fe_3_O_4_ films on MgO(001). The largest effects occur at substrate temperatures of 550 °C and 600 °C. For example, at 550 °C the use of a 1 T field increases the saturation magnetisation from 191 × 10^3^ A/m to 430 × 10^3^ A/m. Together with a five-fold enhancement of the in-plane remanence and an increase of the coercive field from 4.7 mT to 11.8 mT this signals a very large magnetic modification without major changes in the structure, surface morphology, and thickness of the films. The application of a magnetic field immediately after zero-field Fe_3_O_4_ film growth also influences the magnetic microstructure, but the magnitude of the effect is smaller. The ability to drastically alter the magnetic properties of Fe_3_O_4_ films on Al_2_O_3_(0001) and MgO(001) using a large in-plane magnetic field during PI MOCVD growth is unusual. The absence of any significant change in the film crystalline texture, surface morphology, and film thickness indicates that the magnetic field affects the magnetic microstructure but not the chemistry, thermodynamics, and kinetics of the PI MOCVD thin-film growth process. In other words, the origin of the observed field effects is purely magnetic. This contrasts with other attempts to control the magnetic properties of Fe_3_O_4_ using a magnetic field during film growth. For example, Tang et al. [36], have found that the saturation magnetisation of magnetron sputtered Fe_3_O_4_ films increased from 390 × 10^3^ A/m to 420 × 10^3^ A/m upon the application of a 35 mT field. This effect, however, coincided with clear changes in the crystalline orientation and grain morphology. The difference between these experiments is possibly due to the different nature of the magnetron sputtering and PI MOCVD methods, dissimilar growth temperatures (room temperature + annealing versus high temperature deposition), or the strength of the applied magnetic field. 

With respect to temperature, it is interesting to note that the largest magnetic field effects in our samples were obtained at 550 °C and 600 °C under Ar atmosphere and at 500 °C under Ar+H_2_ atmosphere, irrespective of the substrate. For example, in the case of deposited under Ar atmosphere Fe_3_O_4_ films the saturation magnetisation increased with the deposition temperature and was the largest for films deposited at 600 °C (e.g., up to 666 kA/m on Al_2_O_3_(0001) substrates in 1 T field). However, when films were grown under Ar+H_2_ atmosphere the largest magnetisation values were observed for Fe_3_O_4_ nanofilms which were deposited at 500 °C (e.g., up to 842 kA/m on Al_2_O_3_(0001) substrates in 1 T field). These are the films with the highest degree of Fe_3_O_4_ (111) film texture. In all these cases the increase in saturation magnetisation can be explained by the improvement in crystalline quality of the magnetite films and reduction in antiphase boundaries which are occurring during growth of Fe_3_O_4_ thin films. Additionally, the Fe_3_O_4_ films on Al_2_O_3_(0001) contain a minority α-Fe_2_O_3_ phase when grown at 450 °C, 500 °C, or 550 °C, but this phase does not appear in the X-ray diffraction and Raman spectroscopy measurements of the sample deposited at 600 °C or any of the Fe_3_O_4_ films on MgO(001). This clearly demonstrates that the field effects are not related to the presence of a secondary iron oxide phase but instead originate from the Fe_3_O_4_ crystal lattice and the defects (e.g., antiphase boundaries) therein. One possible explanation for a drastic increase of the film magnetisation in an applied magnetic field of 1 T is related to the alignment of paramagnetic spins at high deposition temperatures. If no external field is used, the spins are oriented randomly and during cool down both ferromagnetic and antiferromagnetic ordered regions are established. In a 1 T field, on the other hand, the spins are aligned when the film is cooled through the magnetic ordering temperature and this favours the formation of a ferromagnetic lattice. This hypothesis relies on a large degree of antiferromagnetic order in Fe_3_O_4_ films that are grown without an external magnetic field, which is supported by the relatively small magnetisation values. In thin Fe_3_O_4_ films, an antiferromagnetic spin alignment is not uncommon and is normally associated with defect structures such as antiphase boundaries. It is known that antiferromagnetic and frustrated exchange interactions across antiphase boundaries generally result in lower magnetization [28,29,30,31]. The influence of magnetic field cooling is also confirmed by the data on films that are first grown without an applied magnetic field and subsequently cooled in 1 T. The observation that growth and cooling in a magnetic field results in even larger effects suggests that spin alignment during film growth also plays an important role in establishing ferromagnetic order. 

Finally, we have also noticed that in general the magnetisation of the Fe_3_O_4_ nanofilms increases with the increase in film thickness for films grown at the same temperature with and without an external magnetic field. The increased magnetisation values were also observed for three different film thicknesses when grown in an external magnetic field. The increase in the magnetisation values with film thickness can also be explained by the decrease in disordered Fe_3_O_4_ phase atoms at the grain boundaries and better crystallinity. 

## 4. Conclusions

In this paper, we have demonstrated that Fe_3_O_4_ nanofilms can be successfully grown using a single-step PI MOCVD process using an Fe(tmhd)_3_ precursor. It was found that the films develop a preferred (111) crystalline orientation on Al_2_O_3_(0001) and MgO(001) substrates irrespective of deposition temperature. For films on Al_2_O_3_(0001), however, a secondary α-Fe_2_O_3_ phase was also detected at lower deposition temperatures (≤550 °C), but pure Fe_3_O_4_ films could be grown at 600 °C. The films on MgO(001) were fully epitaxial and of high structural quality with a small compressive strain perpendicular to the film plane. The surface morphology of the Fe_3_O_4_ nanofilms consisted of aggregated grains whose size varied strongly with deposition temperature and substrate. Together with the observed fluctuations in film thickness, this clearly reflected dissimilar optimal precursor decomposition/absorption and diffusion rates on Al_2_O_3_(0001) and MgO(001) substrates.

We have also found that magnetite nanofilms grown in Ar+H_2_ atmosphere have better crystalline quality than those grown in Ar only. In addition to the crystalline texture and surface morphology, the deposition temperature also drastically influenced the magnetic properties of Fe_3_O_4_ films. In general, the remanent and saturation magnetisations were found to increase with temperature, which correlated with an improvement of the crystalline quality and, for Al_2_O_3_(0001), a disappearance of the secondary α-Fe_2_O_3_ phase. Differences between Fe_3_O_4_ films on Al_2_O_3_(0001) and MgO(001) included a smaller coercive field and a larger saturation field on the latter. 

As one of the main results of this work, we have shown that the application of an external magnetic field during PI MOCVD growth of Fe_3_O_4_ nanofilms significantly changes magnetic properties, while the texture, surface morphology, and film thickness remain practically unaffected. On both substrates, large increases in the remanent and saturation magnetisations were observed in films that were prepared in a 1 T field. These effects were particularly large at elevated deposition temperatures of 550 °C and 600 °C under Ar atmosphere and at 500 °C under Ar+H_2_ atmosphere. Moreover, it was found that the application of a 1 T field during sample cool down also improves the magnetic properties. The ability to control magnetic properties of Fe_3_O_4_ films using the application of large magnetic fields during growth opens up new routes towards the fabrication and applications of magnetic thin film devices. 

## Figures and Tables

**Table 1:** Overview of the deposition conditions and Fe_3_O_4_ film thickness.

Deposition temperature	450–600 °C	
Evaporation temperature	140 °C	
Carrier gas flow	80 mL/min	
Carrier gas	Ar, Ar+10% H_2_	
Reactor pressure	5 mbar	
Metalorganic precursors	Fe(tmhd)_3_	
Solvent	1,2-dimethoxyethane	
Fe concentration in solution	0.015 mol/L	
Impulse frequency	2 Hz	
Microdose mass	3 mg	
Substrates	MgO(100), Al_2_O_3_(0001)	
**Deposition Temperature (°C)**	**Thickness (nm)**	
	**Al_2_O_3_(0001)**	**MgO(001)**
Films grown in Ar atmosphere		
450	44	73
450, 1 T	38	67.5
500	~100	140
500, cool down in 1 T	~100	137
500, 1 T	~100	144
550	46	45
550, cool down in 1 T	50	39
550, 1 T	45	31.5
600	40	25
600, 1 T	24.5	21
Films grown in Ar+10%H_2_ atmosphere		
500	30.5	55
500, 1 T	28	52
550	92	134
550, 1 T	89	129

**Table 2:** The magnetic properties of Fe_3_O_4_ films grown on Al_2_O_3_ at different deposition parameters.

Al_2_O_3_	Magnetic Parameters				
	Specific (mass) Magnetisation, (Am^2^/kg)	Saturation Magnetisation, M_S_ (10^3^ A/m) at 1 T Field	Remanence, M_R_ (Am^2^/kg)	Coersive Field, H_C_, (10^−4^ T)	Magnetic Moment per f.u. (μ_B_/f.u.)
**Grown in Ar only atmosphere**					
450	44	227	20	206	1.82
450, 1 T	57	296	29	240	2.37
550	65	337	34	360	2.70
550, cool. 1 T	76	393	45	502	3.15
550, 1 T	100	518	62	640	4.15
600	72	372	40	500	2.94
600, 1 T	130	666	81	705	5.34
**Grown in Ar+H_2_ atmosphere**					
500	152	781	95	520	6.26
500, 1 T	160	841	99	640	6.74
550	35	182	21	480	1.45
550, 1 T	37	189	19	540	1.51

**Table 3:** The magnetic properties of Fe_3_O_4_ films grown on MgO at different deposition parameters.

MgO	Magnetic Parameters				
	Specific (mass) Magnetisation, (Am^2^/kg)	Saturation Magnetisation, M_S_ (10^3^ A/m) at 1 T Field	Remanence, M_R_ (Am^2^/kg)	Coersive Field, H_C_ (10^−4^ T)	Magnetic Moment per f.u. (μ_B_/f.u.)
**Grown in Ar only atmosphere**					
450	16	82	6.8	100	0.66
450, 1 T	21	107	9.3	100	0.86
500	32	166	19.6, (4.8⊥)	83, (185⊥)	1.33
500, cool. 1 T	31	159	18.1, (5.2⊥)	145, (220⊥)	1.27
500, 1 T	35	181	22.2, (6.3⊥)	120, (210⊥)	1.45
550	37	191	5, (6.8⊥)	47, (130⊥)	1.53
550, cool. 1 T	45	233	10	90	1.87
550, 1 T	83	430	31, (24⊥)	118, (185⊥)	3.45
600	57	296	6.1, (11.3⊥)	52, (153⊥)	2.37
600, 1 T	89	457	13.6, (9.1⊥)	35, (60⊥)	3.66
**Grown in Ar+H_2_ atmosphere**					
500	114	592	61	126	4.74
500, 1 T	122	630	70	90	5.05
550	12	63	3.5	89	0.51
550, 1 T	18	71	4.1	68	0.73

## Supplementary Materials

The following are available online at http://www.mdpi.com/2079-4991/8/12/1064/s1, Figure S1: X-ray diffraction scans of Fe_3_O_4_ films grown at different temperatures on Al_2_O_3_(0001). Table S1: The average grain size calculated for Fe_3_O_4_ films grown on Al_2_O_3_ substrate. Figure S2: Phi scan of the (511) reflection for a Fe_3_O_4_ film on MgO(100). Table S2: The FWHM, rocking curve, a lattice parameter, and average grain size values of the Fe_3_O_4_ films on MgO substrate. Figure S3: SEM images of Fe_3_O_4_ films on Al_2_O_3_(0001) substrates grown in Ar atmosphere. The images in the left and right column correspond to films grown without and with a magnetic field, respectively. Figure S4: SEM images of Fe_3_O_4_ films on MgO(001) substrates grown in Ar atmosphere. The images in the left and right column correspond to films grown without and with a magnetic field, respectively. Figure S5: In-plane (left) and out-of-plane (right) hysteresis curves of Fe_3_O_4_ films grown on MgO substrates at 550°C with and without an external magnetic field of 1 T. The blue data points indicate the effect of cooling in an external magnetic field of 1 T.
