# Supplementary material for: Deposition of Magnetite Nanofilms by Pulsed Injection MOCVD in a Magnetic Field
Source: Nanomaterials (Basel). 2018 Dec 17;8(12):1064. doi: 10.3390/nano8121064 (PMC6316053; doi:10.3390/nano8121064)
Supplement: Supplementary file 1 [file nanomaterials-08-01064-s001.pdf]

Supplementary Materials: Deposition of magnetite nanofilms by pulsed injection MOCVD in magnetic field

Anna Zukova, Arunas Teiserskis, Yuliya Rohava, Alexander V. Baranov, Sebastiaan van Dijken and Yurii K. Gun'ko

1. X-Ray diffraction results

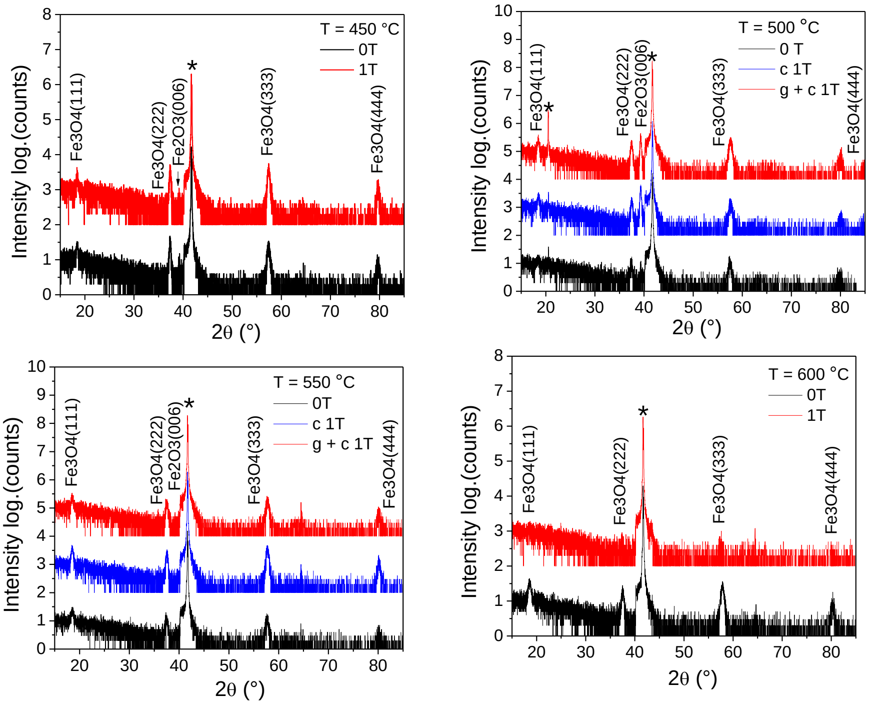

Figure S1. X-ray diffraction scans of Fe_3O_4 films grown at different temperatures on Al_2O_3(0001). In the legend, c indicates that the films were grown without a magnetic field and cooled in 1 T and c + g indicates that the films were grown *and* cooled in a 1 T field.

Table S1. The average grain size calculated for Fe_3O_4 films grown on Al_2O_3 substrate.

Average grain size, (nm)		
**T deposition, (°C)**	**Grown in Ar**	**Grown in H_2+Ar**
450	15.7	—
450, 1T	17.4	—
500	14.2	11.1
500, cooled in 1T	12.7	—
500, 1T	13.4	11.2
550	14.1	12.3
550, cooled in 1T	—	—
550, 1T	15.1	11.3

600	14.5	__
600, 1T	15.7	__

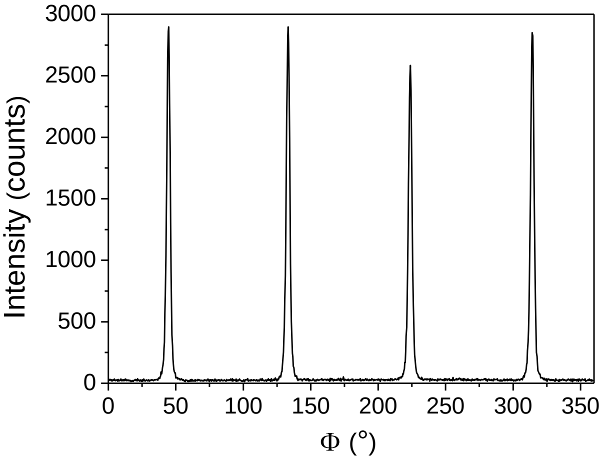

Figure S2. Phi scan of the (511) reflection for a Fe_3O_4 film on MgO(100).[1].

Table S2 The FWHM, rocking curve, *a* lattice parameter, and average grain size values of the Fe_3O_4 films on MgO substrate.

T deposition, (°C)	FWHM, (°)	Omega FWHM, (°)	a lattice parameter, (Å)	Average grain size, (nm)
**Fe_3O_4 grown in Ar atmosphere**				
450	0.66	0.68	8.340	13.7
450, 1T	0.72	0.84	8.337	12.5
500	0.56	0.83	8.318	16.1
500 cooled in 1T	0.53	0.79	8.315	17.2
500, 1T	0.58	0.79	8.312	15.6
550	0.72	0.56	8.301	12.5
550 cooled in 1T	0.69	0.68	8.293	13.0
550, 1T	0.79	0.64	8.302	11.5
600	0.53	0.66	8.284	17.1
600, 1T	0.93	0.68	8.300	9.8
**Fe_3O_4 grown in Ar+H_2 atmosphere**				
500	0.49	0.68	8.362	10.5
500, 1T	0.51	0.71	8.360	11.3
550	0.86	0.69	8.352	18.3
550, 1T	0.80	0.57	8.348	17.9

2. Scanning electron microscopy (SEM) results

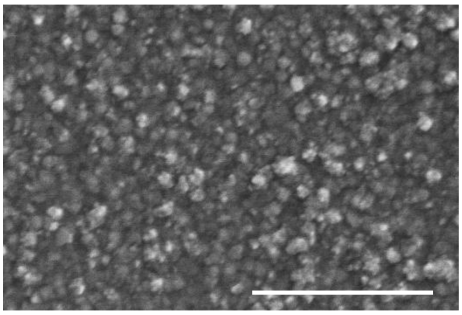
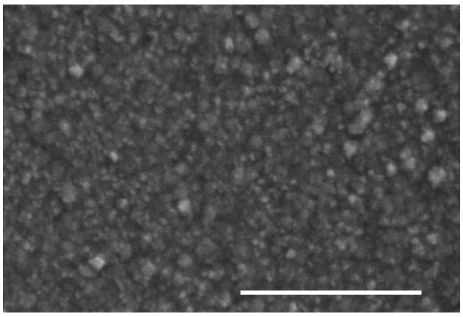

450ºC 450ºC,1T

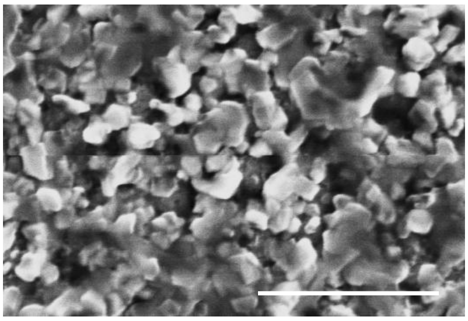
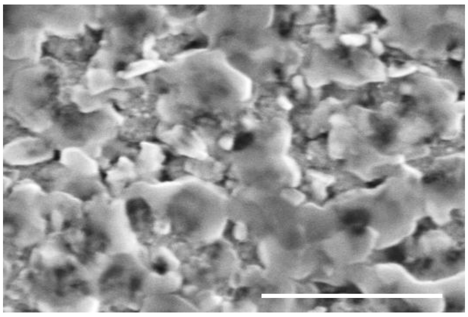

500ºC 500ºC,1T

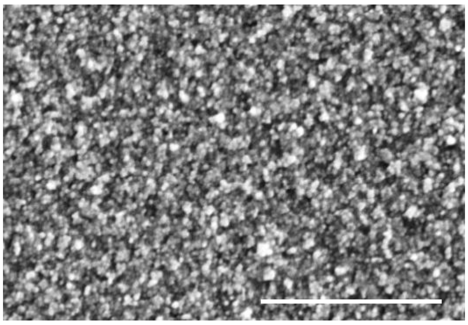
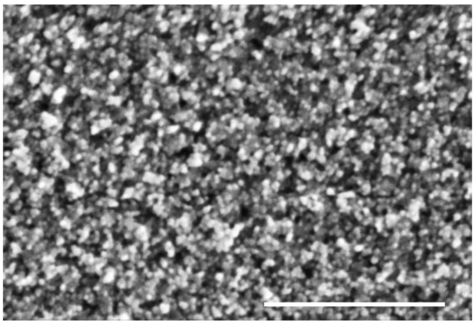

550ºC 550ºC,1T

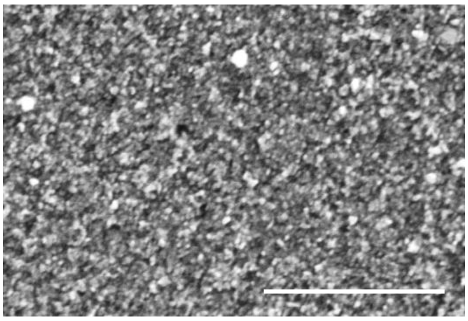
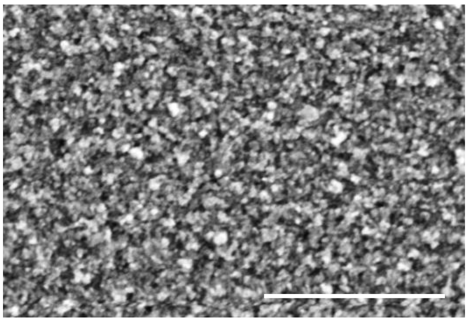

600ºC 600ºC,1T

Figure S3. SEM images of Fe_3O_4 films on $Al_2O_3(0001)$ substrates grown in Ar atmosphere. The images in the left and right column correspond to films grown without and with a magnetic field, respectively. All scale bars are 1μm.

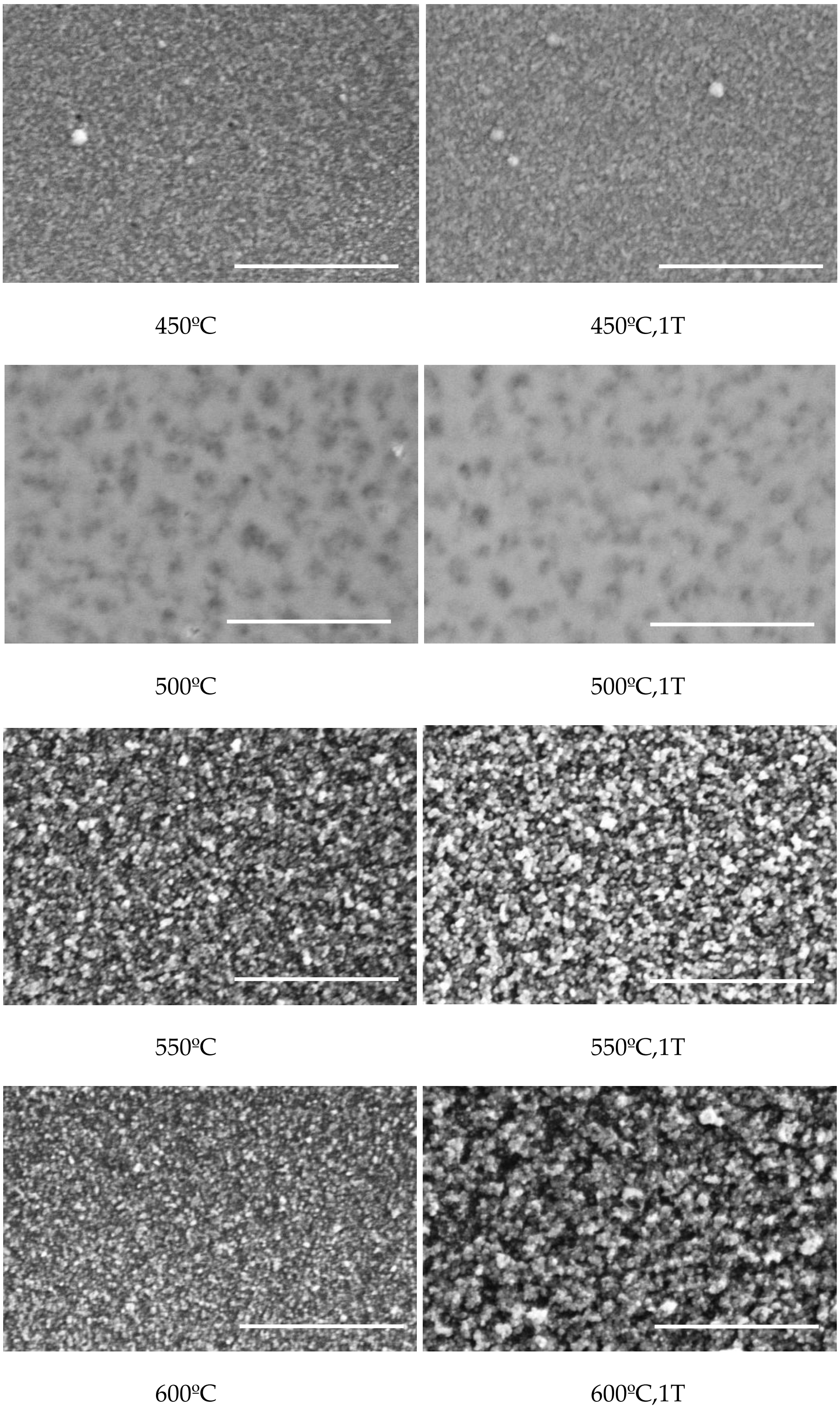

Figure S4. SEM images of Fe_3O_4 films on MgO(001) substrates grown in Ar atmosphere. The images in the left and right column correspond to films grown without and with a magnetic field, respectively. All scale bars are 1μm.

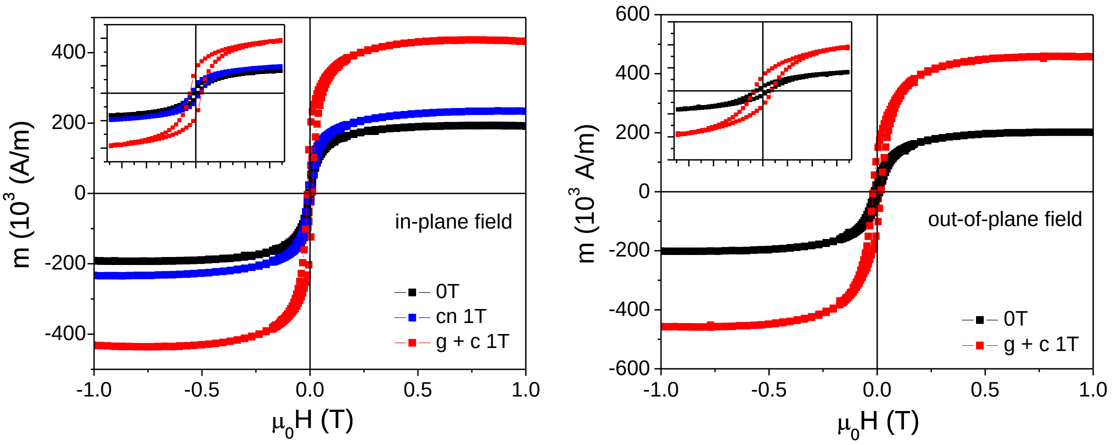

Figure S5. In-plane (left) and out-of-plane (right) hysteresis curves of Fe_3O_4 films grown on MgO substrates at 550°C with and without an external magnetic field of 1 T. The blue data points indicate the effect of cooling in an external magnetic field of 1 T.[1]

References

1. Zukova, A.; Teiserskis, A.; Gun'ko, Y.K.; Sanchez, A.M.; van Dijken, S. Anomalous magnetic field effects during pulsed injection metal-organic chemical vapor deposition of magnetite films. Applied Physics Letters 2010, 96.
